# The emotional map of Prague – data on what locals think about the Czech capital?

**DOI:** 10.1016/j.dib.2021.107649

**Published:** 2021-11-27

**Authors:** Jiří Pánek, Radek Barvíř, Jakub Koníček, Milan Brlík

**Affiliations:** a Department of Development and Environmental Studies, Palacky University Olomouc, Czech Republic; b Department of Geoinformatics, Palacky University Olomouc, Czech Republic; c Prague Institute of Planning and Development, Czech Republic

**Keywords:** Participatory mapping, Emotional maps, Sense of place, Urban geography

## Abstract

The paper presents the data from the participatory emotional mapping in Prague, Czech Republic. It contains 98,364 points complemented with 30,941 comments from 5,973 respondents across the city of Prague (1,335,084 inhabitants according to [1]). There were eight questions/statements common for all of Prague, furthermore each Prague district (*n* = 27) could add up to seven questions/statements. The data were collected via our own participatory mapping platform EmotionalMaps.eu from April to September 2021.

## Specifications Table

**Table:** 

Subject	*Social science – geography*
Specific subject area	*Results from participatory emotional mapping in Prague ranging from safety to transport and free time activities.*
Type of data	Raw data (GeoJSON) accessible at: Panek, Jiri; Barvir, Radek; Konicek, Jakub; Brlik, Milan (2021), “Emotional map of Prague”, Mendeley Data, V1, https://doi.org/10.17632/bmzwzwcw9w.1
How the data were acquired	*Data were collected via participatory mapping web platform from April to September 2021. The survey was in Czech, English translation of the respondents’ comments were created via DeepL Translator.*
Data format	Raw
Description of data collection	*Data were collected via web participatory mapping platform EmotionalMaps.eu. The data collection campaign was open from April to September 2021, when 5973 respondents marked 98,364 locations across Prague. Eight questions were common for all Prague districts. Furthermore, some districts decided to ask additional questions.*
Data source location	*Institution: Prague Institute of Planning and Development* *City: Prague* *Country: Czech Republic*
Data accessibility	Repository name: Mendeley Data Data identification number: Emotional map of Prague Direct URL to data: https://data.mendeley.com/datasets/bmzwzwcw9w/1

## Value of the Data

• Our dataset provides the largest sample of participatory emotional mapping in the Czech Republic so far, and possibly also in Europe. It allows further analysis of the urban sense of place and perceptions across various topics.
• The data can be analysed by researchers focusing on crime geography, transport geography, as well as environmental psychologists and urban planners. The variety of topics is complemented with 30,941 comments about specific points.
• The data provide insights into a broad variety of the citizens´ experiences and perceptions in diverse, areas of interest (free time, places for visitors, neglected places, safety, transport, parking issues, urban green areas, and waste management) with information about the respondents’ age and gender.

## 1 Data Description

**Fig. 1:**
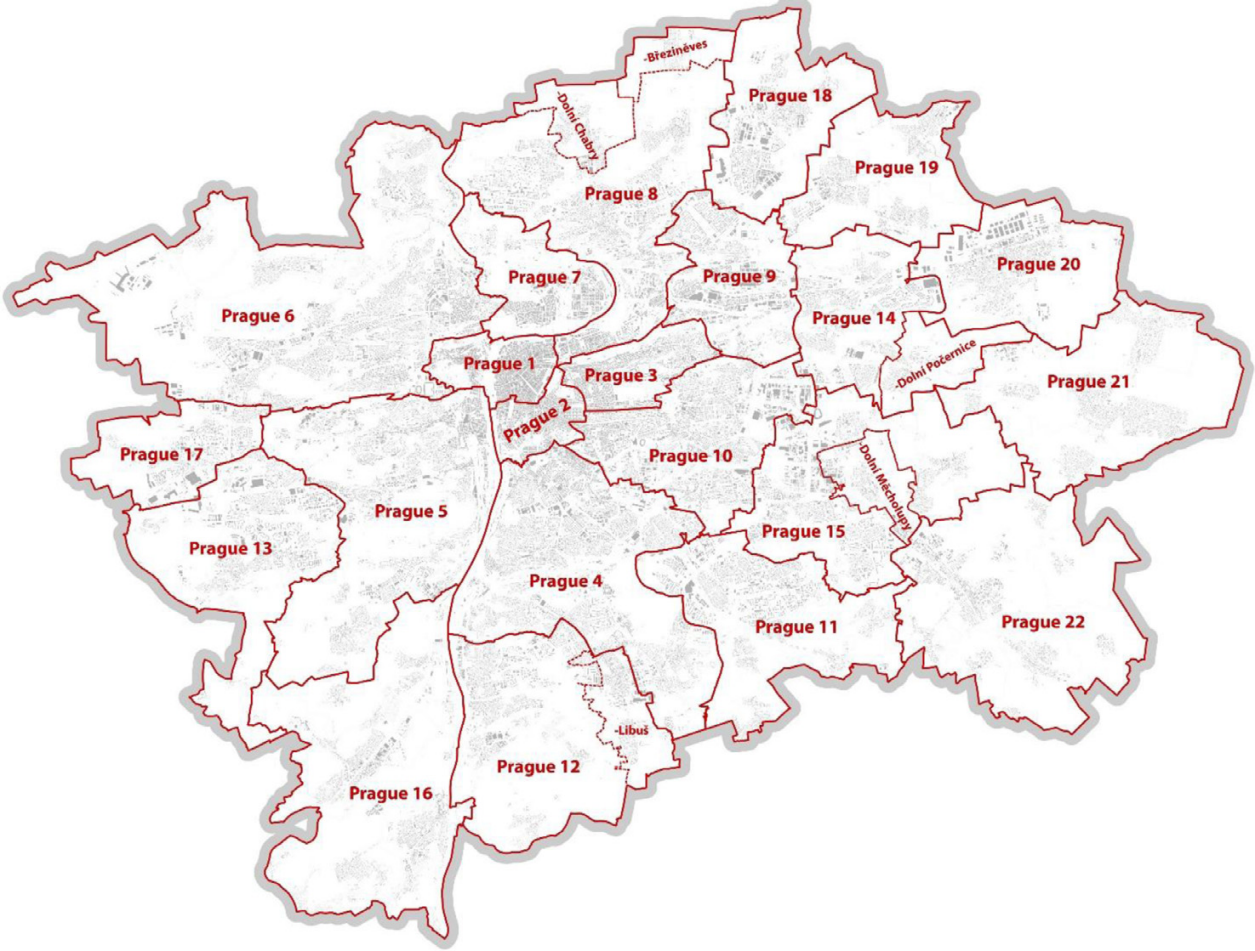
Distribution of Prague districts.

Data about urban perceptions and sense of place are valued amongst researchers from various disciplines, not just geospatial, but also from social sciences and environmental studies. Within our research we created a robust participatory mapping webpage, which included 27 separate emotional maps for each Prague district (Fig. 1), which are the basis for an overall city synthesis of mapped topics. The respondents were first asked to fill in the map for the district where they live, and then they could continue with other districts if needed.

As is visible from Table 1, the sample is not representative regarding the age groups nor the gender balance, but as the data collection method can be defined as open and snowball-like, it was not the aim of this study to have a sociologically representative sample. Nevertheless, we believe it is sizeable enough to provide a complete overview.

**Table 1:** Overview of respondents’ basic demographics.

Age groups	0–19	20–29	30–39	40–49	50–59	60–69	70+	(blank)	Total (excl. empty replies)	Total (incl. empty replies)
Number of respondents	3302	21,133	39,014	20,718	6987	3170	1065	2975		98,364
Women	936	9548	17,146	9738	4020	1870	661	N/A	44,571	N/A
Women in%	28.3%	45.2%	43.9%	47.0%	57.5%	59.0%	62.1%	N/A	46.3%	N/A
Men	2366	11,533	21,776	10,932	2967	1251	404	N/A	51,607	N/A
Men in%	71.7%	54.8%	56.1%	53.0%	42.5%	41.0%	37.9%	N/A	53.7%	N/A

The presented data were collected during the end of the third Covid-19 wave in the Czech Republic, so the preferred data collection method was the online approach. Physical meetings were originally planned in order to enrich the data collection strategy, nevertheless we were forced to abandon this plan. The dataset contains the raw, unprocessed data, with the exception that the labels and comments were translated into English.

## 2 Experimental Design, Materials and Methods

The data were collected via online participatory mapping platform EmotionalMaps.eu (in Czech) from April to September 2021. The platform was created in 2014 [2] (well described in [3,4] and since then it was used in over 200 various emotional mapping projects in several European countries. The data were collected for each of the Prague city districts separately and then merged. Eight questions/statements were common for all respondents (see Table 2) and then some districts decided to add further questions related to their specific neighbourhood (see Table 3). The eight questions/statements common for all of Prague were decided centrally by representatives of Prague City Hall and The Prague Institute of Planning and Development (IPR Prague). Furthermore, the option to add questions relevant to respective Municipal districts, was offered. We are not aware of the reason(s) why some districts did (not) use the opportunity. Some Prague districts used similar questions (we were not able to monitor or adjust the questions suggested by the districts’ representatives), so the suggested broader categories are presented in Table 4. We do not claim that data from these categories are mutually fully complemented, but we believe they can be used for cross district comparisons if needed. The categories are just suggestions and we are sure users can also find their own categories. The data were collected as point features, mainly based on the argument of [5], who states that “The use of points for mapping PPGIS attributes and aggregating areas through density mapping constitutes a conservative approach to spatial inferences about a places significance, but the data demands for point collection are considerably higher than for polygon features.” In our visualisations we expected users’ inaccuracy in point placement and we used hexagons to aggregate the points into a regular grid for further visualisations. The hexagonal grid nevertheless is not part of the raw data provided in the dataset.

**Table 2:** Common questions/statement for all Prague districts.

Number of the Question/Statement in the dataset	Question/Statement
1	This is where I spend my free time
2	I would show this place to a visitor
3	This place is neglected and needs to be renovated
4	I don't feel safe here (suspicious people, neglected environment, etc.)
5	There is a traffic hazard here (for walking, cycling, motor vehicle, lack of pedestrian crossing etc.)
6	There are parking problems (not enough parking spaces, cars parked inappropriately, etc.)
7	I would like more green space here
8	There is often an overflowing waste bin or collection point for municipal/sorted waste

**Table 3:** Additional questions/statement and how they were used in different Prague districts.

Number of the Question/Statement in the dataset	Question/Statement	Prague district covered with this question/statement
9	What needs to be fixed?	Prague Dolní Chabry
10	Where would you place the bins and dog waste bins?	Prague Dolní Chabry
11	Where is it impossible to get to?	Prague Dolní Chabry
12	Where would you like to make a playground or other public space?	Prague Dolní Chabry
13	Where are you proud of our borough?	Prague Dolní Chabry
14	Where do you suggest changes?	Prague Libuš
15	Where do you feel comfortable?	Prague Libuš
16	Where do you play sports?	Prague Libuš
17	Which stretches of road do you think are dangerous?	Prague Dolní Chabry
18	Which section of the bike path do you think is dangerous?	Prague 21
19	I like to come here	Prague 10
20	This is my favourite public space to use	Prague Březiněves
21	I consider this place to be the centre of our neighbourhood	Prague 10
22	There could be a playful element for children here	Prague Březiněves
23	There could be a pedestrian crossing here	Prague 13
24	There could be a hopscotch or other 2D playful element for children	Prague 13
25	There could be a bench here	Prague Březiněves
26	A cycle path or cycle lane could lead here	Prague 12
27	A cycle path/footpath could lead here	Prague Dolní Měcholupy
28	New trees or an avenue could be planted here	Prague Dolní Počernice
29	I would like a climbing wall here	Prague Dolní Počernice
30	I would appreciate enhanced public transportation here (more frequent connections, new stops, etc.)	Prague 11
31	I would like to see surveillance by the City's CCTV system here (frequent vandalism, theft, obscure places, etc.)	Prague 10
32	I would welcome a dog playground here	Prague Dolní Počernice
33	I would welcome a disabled parking space here (due to frequent visits to the site)	Prague 10
354	I want new services here	Prague Březiněves
35	There is a lack of cycle links here	Prague Dolní Počernice
36	There is a lack of a playground here	Prague 18
37	There is a lack of a bench here	Prague 18
38	There is a lack of a large-capacity car park	Prague 18
39	There is a lack of a multi-purpose playground for the public	Prague Dolní Počernice
40	Here is the centre of our neighbourhood	Prague 11
41	Here there is often a mess	Prague 11, Prague 18, Prague Dolní Měcholupy
42	There is frequent clutter here	Prague Březiněves
43	There is a missing pedestrian crossing	Prague 12
44	This place is alive (good atmosphere)	Prague 11
45	Here is a dangerous place on the cycle path	Prague 18
46	Here is a problematic place to access (barrier)	Prague 10
47	Here is a space where I want to educate myself	Prague Březiněves
48	Here is too much noise	
49	Here is too much noise (from traffic, restaurant, playground, etc.)	Prague 10
50	It's unbearably hot here in summer	Prague 11
51	Here is a good place for a patisserie/café	Prague Dolní Počernice
52	I miss wheelchair access here	Prague 21
53	I miss a bench here	Prague 21
54	I miss a bin here (for mixed waste and dog excrement)	Prague 21
55	I miss street lighting here	Prague 21
56	I miss a pedestrian crossing here	Prague 21
57	I miss sports facilities here	Prague 21
58	Here I suggest a change (playground, green space, water feature, bench or other furniture, workout, artwork, etc.)	Prague 12
59	I don't like to walk here	Prague Březiněves
60	I miss play elements for children here	Prague Dolní Počernice
61	I like to play sports here	Prague 11, Prague Dolní Měcholupy
62	I find it hard to breathe here (air pollution from traffic, industry, etc.)	Prague 10
63	The environment here has deteriorated in the last year	Prague 12
64	The environment here has improved in the last year	Prague 12
65	The air here is bad to breathe and/ or polluted	Prague 12
66	This is where we have our reunions	Prague 11

**Table 4:** Suggested broader categories for questions across the Prague district boarders.

Group number	Questions included	Group topic
1	1, 15, 16, 19, 20, 61	Free time/sport
2	2, 13, 44	Proudness
3	3, 9, 14, 41, 42	Need for change
4	4, 31, 59	Safety
5	5, 6, 11, 17, 18, 23, 26, 27, 30, 33, 35, 38, 43, 45, 46, 52, 56	Transport
6	7, 28	Green space
7	8, 10, 54	Waste bins
8	21, 40	Centre of the neighbourhood
9	12, 22, 24, 29, 36, 39, 57, 60	Children and playgrounds
10	25, 37, 53	Benches
11	48, 49	Noise
12	62, 65	Bad air
13	32, 34, 47, 50, 51, 55, 58, 63, 64, 66	Mix

Table 5 presents basics statistics for eight common questions for all of Prague, including the number of comments for each question. The most commented on (in relative numbers) is the question about traffic hazards, while the least commented on (again in relative numbers) is the statement “I would show this place to a visitor”. The number of points represents how many locations were marked in each question. Each respondent had the opportunity to mark more points in each question and it was possible to skip questions, where the respondent did not want to mark any location.

**Table 5:** How many points and comments was collected in common questions?

Question/statement	1	2	3	4	5	6	7	8
Number of points	15,989	13,283	13,993	11,376	9786	7983	10,416	5514
Number of comments	4274	2746	5330	4051	5561	2917	2357	1387
% of points with comment	26.7%	20.7%	38.1%	35.6%	56.8%	36.5%	22.6%	25.2%

The dataset is saved and shared as a GeoJSON file, an open format for encoding and representing a variety of geographic data structures and features, including their non-spatial attributes (in our case, gender, age, and comments). GeoJSON is an extension of JSON format and can be easily read by any GIS software (we worked with open source QGIS) and any text editors such as Notepad. In the file, each line represents one feature (point), while columns represent the attributes. Coordinates and geometry type are directly written in the structure of the feature, while attribute values are written as “properties” – see below.

 *One feature would be written as this example:{"type": "Feature", "properties": {"ID": 1, "question": 1, "user_id": "d9w9tu8me", "comment_CZ": null, "comment_EN": null, "gender": "male", "age": "30–39″}, "geometry": {"type": "Point", "coordinates": [14.395566, 50.090879]}},*

Columns used in the GeoJSON file of our data are reported below:

• ID – unique Id of each feature
• question – a number that represents the question/statement asked (see Tables 2 and 3 for explanations)
• user_id – unique Id assigned to every user, so one can track answers from a specific user
• comment_CZ – original comments attached to the specific point in the Czech language
• comment_EN – translated comments (we used service DeepL for the translations)
• gender – binary variable – male/female (or empty)
• age – variable that can contain one of these age groups (0–19, 20–29, 30–39, 40–49, 50–59, 60–69, 70+, empty)

## CRediT authorship contribution statement

**Jiří Pánek:** Conceptualization, Methodology, Validation, Writing – original draft, Supervision, Project administration, Funding acquisition. **Radek Barvíř:** Data curation, Writing – review & editing, Visualization. **Jakub Koníček:** Data curation, Writing – review & editing, Visualization. **Milan Brlík:** Data curation, Writing – review & editing.

## Declaration of Competing Interest

The authors declare that they have no known competing financial interests or personal relationships that could have appeared to influence the work reported in this paper.

## Appendix Supplementary materials

Image, application 1
